# The formation and extinction of fear memory in tree shrews

**DOI:** 10.3389/fnbeh.2015.00204

**Published:** 2015-07-29

**Authors:** Shujiang Shang, Cong Wang, Chengbing Guo, Xu Huang, Liecheng Wang, Chen Zhang

**Affiliations:** ^1^State Key Laboratory of Membrane Biology, School of Life Sciences; PKU-IDG/McGovern Institute for Brain Research, Peking UniversityBeijing, China; ^2^Department of Physiology, Anhui Medical UniversityHefei, China

**Keywords:** tree shrew, fear conditioning, memory formation, memory extinction

## Abstract

Fear is an emotion that is well-studied due to its importance for animal survival. Experimental animals, such as rats and mice, have been widely used to model fear. However, higher animals such as nonhuman primates have rarely been used to study fear due to ethical issues and high costs. Tree shrews are small mammals that are closely related to primates; they have been used to model human-related psychosocial conditions such as stress and alcohol tolerance. Here, we describe an experimental paradigm to study the formation and extinction of fear memory in tree shrews. We designed an experimental apparatus of a light/dark box with a voltage foot shock. We found that tree shrews preferred staying in the dark box in the daytime without stimulation and showed avoidance to voltage shocks applied to the footplate in a voltage-dependent manner. Foot shocks applied to the dark box for 5 days (10 min per day) effectively reversed the light–dark preference of the tree shrews, and this memory lasted for more than 50 days without any sign of memory decay (extinction) in the absence of further stimulation. However, this fear memory was reversed with 4 days of reverse training by applying the same stimulus to the light box. When reducing the stimulus intensity during the training period, a memory extinction and subsequently reinstatement effects were observed. Thus, our results describe an efficient method of monitoring fear memory formation and extinction in tree shrews.

## Introduction

Fear is one of the strongest emotions in living entities, arising from the perception of danger or risk in the environment. Fear memory is formed to help animals to avoid harm from a specific stimulus and to adjust their adaptive behavior. Alteration of fear memory formation and erasure is a fascinating subject for researchers, because it will potentially help to optimize the therapies for panic disorder and posttraumatic stress disorder (Hermans et al., 2006). A mounting number of studies on rodents, including mice and rats, have been performed to reveal underlying mechanisms both at the molecular and circuit levels. In rodents, the classical fear conditioning memory can be produced by an intense foot shock and retained for months (Stanton, 2000; Maren et al., 2013).

Classic works have found that the amygdala's microcircuits—including heterogeneous nuclei—and a part of the striatum control learned fear (Davis, 1992; Ehrlich et al., 2009; Pape and Pare, 2010; Duvarci and Pare, 2014). The main site of neuroplasticity, which mediates fear learning, has been reported to be the lateral nucleus of the amygdala (LAn). During fear learning, LAn neurons connecting with cortical synapses and the auditory thalamus are strengthened. Although synapses in the LAn receiving the conditioned stimulus (CS) input might be a promising candidate (Shumyatsky et al., 2002; Zheng et al., 2007; Kwon and Choi, 2009; Park and Choi, 2010), the exact synapses where the information of CS and unconditioned stimulus (US) are integrated and produced still remains a mystery.

Memory extinction is a process in which animals replace previously formed memories with new ones, which is a positive way for animals to adjust their behaviors in response to changed environmental cues (Hong et al., 2011; Maren, 2011; Orsini and Maren, 2012). Typically, memory extinction occurs when the CS is presented in the absence of the US (Joels and Lamprecht, 2014). Thus, extinction training may lead to a new form of memory that inhibits but does not erase the original memory (Bouton et al., 2006; Myers and Davis, 2007; Duvarci and Pare, 2014). Similar to fear memory formation, neuronal circuits in the amygdala are required to mediate the extinction of fear memory (Cammarota et al., 2005; Sotres-Bayon and Quirk, 2010; Sierra-Mercado et al., 2011; Jasnow et al., 2013; Duvarci and Pare, 2014). Other reports have shown that this extinction is also controlled by a series of networks, including the hippocampus and medial prefrontal cortex (Herry et al., 2010; Pape and Pare, 2010; Milad and Quirk, 2012; Maren et al., 2013; Duvarci and Pare, 2014; Herry and Johansen, 2014).

On the other hand, few studies have been performed to investigate the mechanisms of fear memory in higher animals such as nonhuman primates. This is largely due to the lack of appropriate models in higher animal study. In addition to the apparent ethical issues, high costs and limited resources are two major bottlenecks. Tree shrews are small mammals living mainly in Southeast Asia, including India, China, Indonesia, and the Philippines. Molecular phylogeny studies, together with whole genome sequencing analysis, suggest that tree shrews are closely related to primates (Xu et al., 2012, 2013a; Fan et al., 2013; Zhou et al., 2015). Tree shrews have the highest brain–body mass ratio among all animals and display many higher-level activities. For example, tree shrews have been used to study alcohol tolerance, hepatitis B virus infection, psychological stress, depression, cognitive learning, and social learning abilities (Cao et al., 2003; Fuchs, 2005; Yang et al., 2005; Wang et al., 2011, 2012, 2013a; Xu et al., 2013b; Shen et al., 2014). However, tree shrew models related to fear memory formation and extinction have not been reported. Therefore, we investigated whether a conditioning avoidance paradigm could allow the researchers to monitor the formation and extinction of fear memory in tree shrews. We present data demonstrating that tree shrews show learning to avoid harmful stimuli (voltage foot shock, two to five rounds of training) in a light/dark apparatus. The established fear memory lasted for more than 50 days without any sign of extinction when five rounds of training were applied. Memory extinction was observed when two rounds of weaker training were applied. Thus, our data suggest that tree shrews could serve as a valuable model for studying fear memory induced by associative training.

## Materials and methods

### Animals

Adult male tree shrews (*Tupaia belangeri chinensis*) were obtained from the Kunming Institute of Zoology (Kunming, China) and housed in large cages (40.0 × 38.0 × 34.0 cm), which were connected to a nesting box (36.2 × 15.8 × 20.0 cm) through a door. Animals (age: 2–3 years, weight: 120–170 g) were reared in single cages at a temperature- (T) and relative humidity (RH)-controlled room (T: 22–25°C, RH: 55–75%); they were maintained on a standard 12 h light/dark cycle (light on at 07:00) at the Laboratory Animal Center of Peking University. Food and water were provided *ad libitum*. Animals included in the experiment were naive to the current test and had no prior experience in any behavioral test. All animal studies were conducted at the AAALAC-approved Animal Facility at Peking University. Experiments were undertaken in accordance with the guide for the care and use of laboratory animals (eighth edition). All experimental protocols were approved by the Institutional Animal Care and Use Committee of Peking University.

### Chamber design for tree shrews

**Figure 1:**
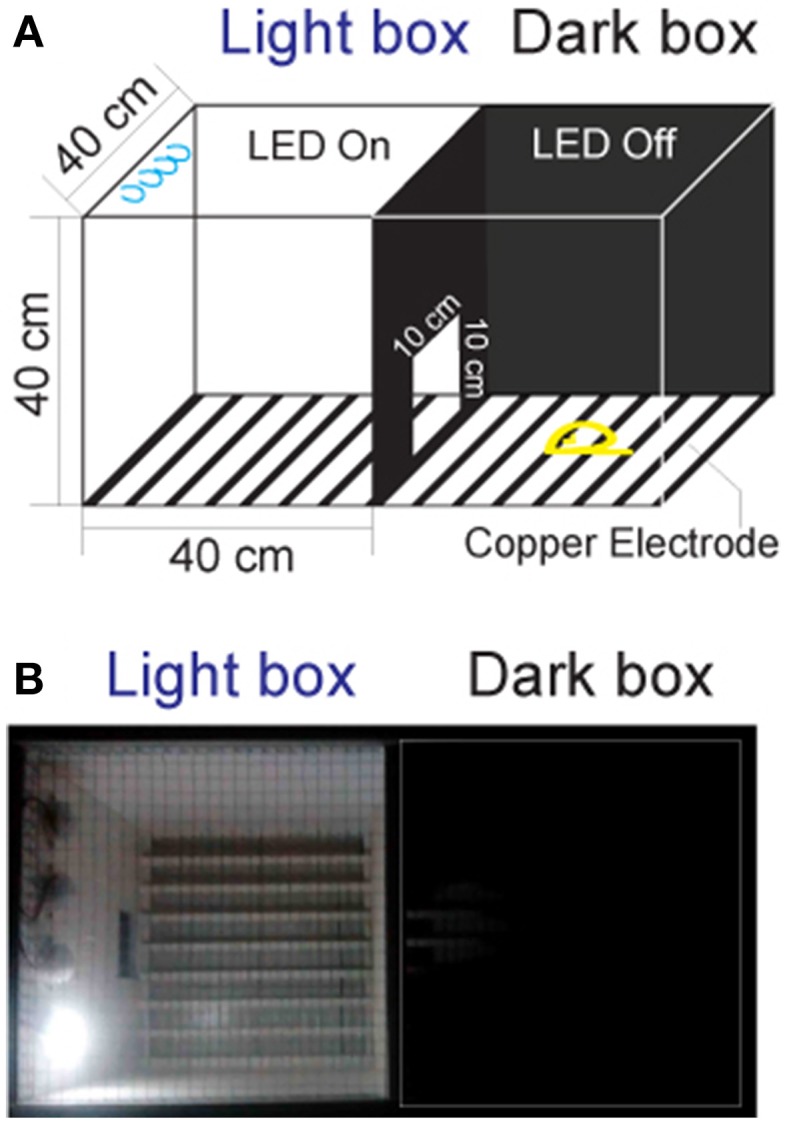
**The apparatus for the fear-conditioning model of the foot shock experiment**. **(A)** The apparatus design. The box in which the LED was turned on was called the “light box,” while the one in which the LED was off was called the “dark box.” Animals could move freely between the two chambers through a door. **(B)** Picture of the apparatus.

Currently, there are no commercially available instruments for studying fear conditioning in tree shrews. The classic systems are designed for rodents, and are not suitable for tree shrews because of their size and motion characteristics. Hence, we constructed the testing apparatus (Figure 1A) using two observation chambers and a video camera (EasyN) fixed 130–150 cm above the top of the apparatus.

Distinct from the classic chambers for rodents (Bourin and Hascoët, 2003; Chang et al., 2009), the dimension of the chambers for tree shrews were set to 40 × 40 × 40 cm, which closely resembled the home cage. The tops of the chambers were made of the stainless steel mesh to prevent the animals from escaping during the experiments. The walls of the chambers were constructed of polypropylene homopolymer intended for injection molding. The floors of the chambers consisted of 23 copper rods (1.2 mm in diameter) spaced 15 mm apart (center to center). All copper rods were connected to an electrical source (Victor) for the delivery of voltage shock (0–30 V, 0.0–2.3 mA). A door (10 × 10 cm) was constructed on the wall between the two chambers to allow the tree shrews to pass through freely. A light-emitting diode (LED, Philips) mounted on the back wall of each chamber was used for turning the designated chamber into either a light or dark chamber.

### Behavior test for tree shrews

The animal behavior was monitored between 14:00 and 18:00, since tree shrews are diurnal mammals. The animals were first transported to the dual-chamber apparatus and allowed to explore freely for 3 days (30 min per day) for arena familiarization. The animals were handled one by one, and the chambers are cleaned with 75% ethanol after each operation. After familiarization, animals were tested in various contexts. Each animal was given a 3 min period to acclimate to the conditioning apparatus with the room lights on before recording. During the experiments, all room lights and LEDs were turned off except for the light box with the LED on (Figure 1B). The light chamber was the chamber where the animal was located at the end of the 3 min acclimation period.

### Data collection and analysis

The behavior of animals was videotaped with an infrared camera (1280 × 720 pixels, 25 frames per second), and the movie was analyzed using EthoVision XT 9 (Noldus). The location of an animal in the dual-chamber was determined by the center of the animal. Data were statistically analyzed using SPSS 16.0.

## Results

### Tree shrews preferred to stay in the dark box with low mobility in the light/dark apparatus

**Figure 2:**
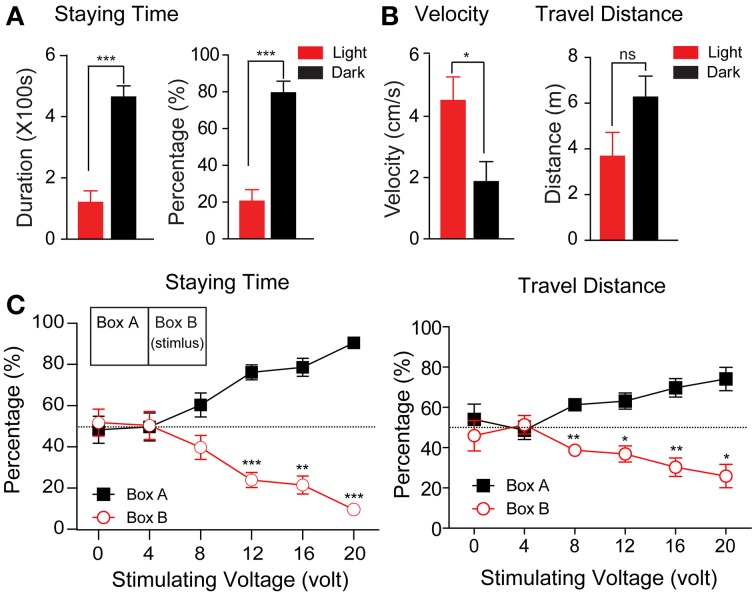
**The statistical results of the regular activity experiment and behavior responses to different voltage levels of foot shock. (A,B)** The absolute values and percentage of the staying time **(A)** and velocity and travel distance **(B)** were calculated during the test phase (*n* = 8, ^*^p < 0.05, ^***^p < 0.001). The data from this experiment were analyzed using a paired-samples *t*-test. **(C)** The percentage of staying time (left) and travel distance (right) in each box during the test phase were plotted in relation to the voltage levels of the foot shock (*n* = 6, ^*^p < 0.05, ^**^p < 0.01, ^***^p < 0.001). The stimuli were delivered as depicted in the inset (right panel). The data from this experiment were analyzed using a paired-samples *t*-test. All data were expressed as mean ± standard error of the mean (SEM). Some brackets are not visible because the SEM was smaller than the data point.

We first tested the tree shrews' behavior in the context of the light/dark apparatus. After familiarization with the apparatus, the locomotor activities of the animals were recorded for 10 min each day for 4 days. As shown in Figure 2A, tree shrews spent significantly less time in the light box, whether calculated by absolute values (Figure 2A, left, light box: 120.3 ± 37.50 s; dark box: 463.9 ± 36.57 s; *p* < 0.0001) or percentages (Figure 2A, right, light box: 20.51 ± 6.31%, dark box: 79.49 ± 6.31%, *p* < 0.0001). Tree shrews were less mobile in the dark box, as reflected in the significantly lower mean velocity (Figure 2B, light box: 4.52 ± 0.77 cm/s, dark box: 1.87 ± 0.66 cm/s, *p* < 0.05). Thus, the travel distance was not significantly different, although it was slightly reduced in the light box (Figure 2C, light box: 367.5 ± 104.5 cm, dark box: 626.1 ± 92.21 cm, *p* > 0.05).

### The tree shrews showed avoidance of the foot voltage shock depending on the intensity of the stimulus

In associative learning, an animal predicts the US by perceiving the conditioned stimulus CS(Byrne, 1987; Wasserman and Miller, 1997; Fanselow and Poulos, 2005; Correa, 2007). The US can be either a reward or a noxious reaction. Here, we tested whether a voltage shock applied to the footplate could serve as a noxious US to induce fear in tree shrews. The animals were placed in the apparatus with the light on in both chambers. Voltage pulses (10 s stimulus with a 30 s interval for a total of 10 min) ranging from 0 to 20 V in voltage (equivalent to 0.0 to 1.3 mA in currents, Supplemental Figure 1) in 4 V steps were applied to one of the two boxes. As a control condition, the animals spent almost equal amounts of time in both boxes when no voltage pulses were given to the footplate (48.22 ± 6.58% and 51.78% ± 6.58%; *p* > 0.05).

As shown in Figure 2C, the animals reduced the percentage of their staying time in the stimulating box when the stimulus voltage levels were increased. At 4 and 8 V, the percentage of time the animal spent in the stimulating box showed a declining trend, although this was not significantly different from 50% (50.36 ± 6.79 and 39.70 ± 5.84%, respectively). At 12, 16, and 20 V, the percentage was significantly reduced to 23.88 ± 3.62, 21.39 ± 4.36, and 9.56 ± 2.01%, respectively (*p*_12V_ < 0.001, *p*_16V_ < 0.01, *p*_20V_ < 0.0001). Similarly, the percentage of travel distance was also reduced with increasing stimulus intensity (12 V: 36.87 ± 3.97%, *p* < 0.05, 16 V: 30.32 ± 4.58%, *p* < 0.01; 20 V: 25.91 ± 5.81%, *p* < 0.01). These data clearly show that a foot shock with voltage exceeding 12 V induced significant avoidance behavior in tree shrews, and was an effective noxious stimulus for the animals in the designed apparatus.

### Foot shocks effectively induced the formation of fear memory in tree shrews

Long-term fear memory could be formed when the conditioned sensory stimulus is delivered with an unconditioned scaring event (Hong et al., 2011; Johansen et al., 2011; Herry and Johansen, 2014). Based on the avoidance behavior of tree shrews in our previous experiments, we first used voltage shock (16 V, equivalent to 1.05 mA in currents) to test whether this would change the behavior of the animals in the light/dark apparatus and whether this behavior change (memory) turned into a long-term memory. The experimental design was composed of six stages, as follows: adaptation, baseline, training, monitoring phase 1, a reverse training period, and monitoring phase 2. Animals were randomly divided into a control group (*n* = 4, without stimulus) and a trial group (*n* = 4, with stimulus). After arena familiarization for adaptation, the activities of tree shrews were recorded in the light/dark box for 4 days to establish the baseline.

**Figure 3:**
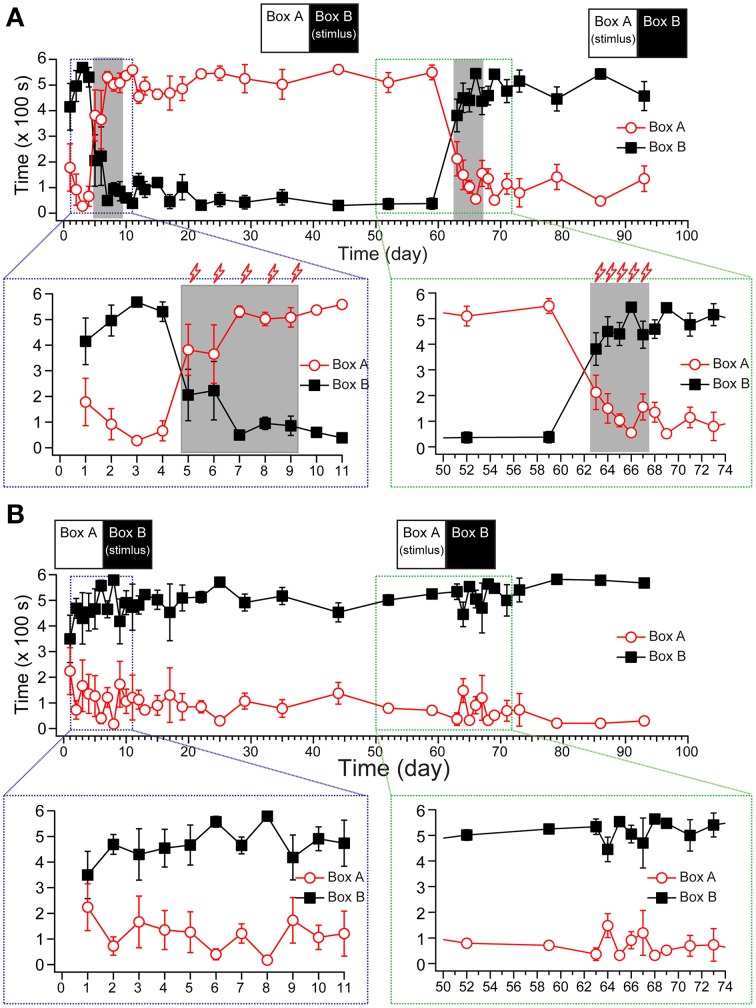
**The establishment of fear-conditioned memory in tree shrews**. The statistical results show the staying time of the trial group (**A**, *n* = 4) and the control group (**B**, *n* = 4) from pre-stimulus recording to the end. The stimuli were delivered as depicted in the inset (top). A zoomed projection (bottom) shows the process of memory establishment (left) and reverse learning (right). All data were expressed as mean ± SEM. Some brackets are not visible because the SEM was smaller than the data point.

As shown in Figure 3A, the tree shrews spent 502.3 ± 29.73 s of time in the dark box and 91.02 ± 29.90 s in the light box during the baseline recording period. Then, the voltage stimuli (16 V stimulus for 10 s, with a 30 s inter-stimulus interval [ISI], 10 min of training per day) were delivered to the dark box for 5 days to induce the formation of fear memory in the trial group. Our data showed that the animals spent significantly less time in the stimulating box (the dark box in the first training stage), even after the first day of stimulus, and the avoidance behavior reached a plateau during the third to fifth day of stimulus.

Using a post hoc Duncan's multiple range test with Huynh/Feldt adjustment to analyze the within-group effect, the animals significantly changed their preference for the light/dark box compared with the baseline period within 3 days {Figure 3A, [*F*_(34, 204)_ = 9.757, *p* < 0.001, *n* = 4] for the light box; [*F*_(34, 204)_ = 10.560, *p* < 0.001, *n* = 4] for the dark box}. To test whether the fear memory had turned into long-term memory, we monitored the activity of tree shrews in the same apparatus without foot shock (US) for 51 days (10 min recordings, once per day for post-training days 1–5, once every 2 days for days 6–13, once every 3 days for days 15–26, and once every 7 days for days 29–51 after the stimulus). The percentage of staying time in the dark box remained stable during the 51 days of recordings after training period, suggesting that the new memory did not recede during the post-stimulus recording phase.

Next, we applied the same daily stimulus to the light box for 4 days to test whether this would reverse the pre-obtained memory from the first training. Our results showed that reverse training for 4 days (days 63–66) rapidly reversed the animals' preference for the light box that had developed in the first training stage. We monitored the animals' locomotor activities for 10 additional days after the reverse training, and found that the percentage of activity in the dark box remained stable when compared with the last day in the reverse training stage (Figure 3A), suggesting that the previous fear memory was erased, and a new memory had been successfully established. The control group, which was treated the same as the trial group except that the voltage stimulus in the training and reverse training periods was not applied, showed reliable behavior performance in the light/dark box during the 3 month experimental period (Figure 3B).

### Observation of memory extinction and reinstatement effects in tree shrews

Memory extinction is observed in animals when the CS is no longer associated with the US. As shown in Figure 3, the tree shrews exhibited almost no extinction of fear during the 51 days of repeated monitoring after training. In addition, after reversal learning, the newly obtained memory was not subject to extinction during the 10 days of post-conditioning monitoring.

**Figure 4:**
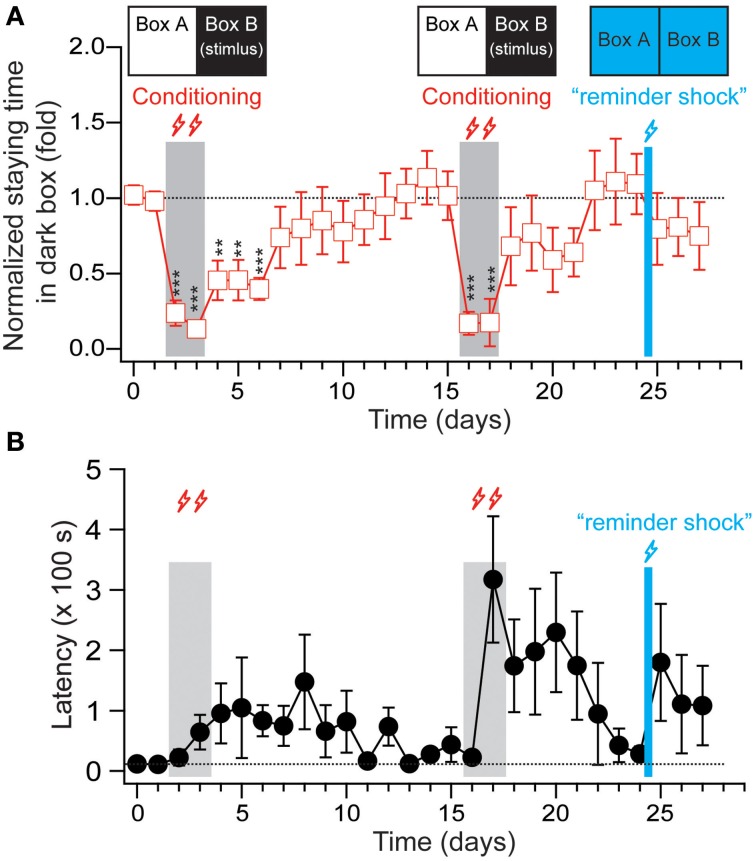
**The extinction and reinstatement effects of fear-conditioned memory in tree shrews. (A)** The staying time of the animals in the dark box (stimulating box in the conditioning training) were normalized to the average staying time during the two-days pre-conditioning period and plotted versus the recording days. The “reminder shock” delivered at Day 25 is 12 V stimulus for 10 s with 60 s ISI applied to one of the chambers for 5 min, and the other one for another 5 min. During the stimulation, the room light was kept on and chamber LEDs was kept off. **(B)** The latencies of the animals entering the dark box for the first time during each recording session were plotted from the same experiment depicted in **(A)**. All data were expressed as mean ± SEM. The data were analyzed using a paired-samples *t*-test. *n* = 7, ^**^p < 0.01, ^***^p < 0.001.

After normalization, the percentage of staying time in the stimulating chamber declined by 1.1 ± 0.4 and 2.3 ± 1.3% per day in the post-conditioned period after the training and reverse training period, respectively (Supplemental Figure 2). This may have been partially caused by the strong stimuli used in the experimental paradigm, which were close to the saturation level. Thus, we adopted a weaker shock (12 V/0.79 mA stimulus for 10 s, with 60 s ISI, with 10 min of training per day, for 2 days) to induce fear memory. As shown in Figure 4, this weaker stimulation successfully changed the animals' staying time in the stimulating chamber from 331.24 ± 40.72 s to 42.12 ± 14.68 s (*p* < 0.01, *n* = 7). We then monitored whether the animal show the extinguish of the avoidance behaviors for an additional 7 days, and found that the staying time in the post-conditioning period declined by 8.1 ± 2.8% per day and returned to the pre-conditioning baseline within 7–10 days. After the extinction was almost complete at Day 15, the tree shrews were given another same 2-day foot shock. The results show an even fast extinction rate (11.3 ± 2.7% per day) in the absence of US. These observations clearly indicate an extinction process of memory in tree shrews.

When extinction is observed, a “reminder shock” (12 V stimulus for 10 s with 60 s ISI applied to one of the chambers for 5 min, and the other one for another 5 min; with room light on and chamber LEDs off) was delivered to stimulate the animals. Following this, the tree shrews were put back in the context of a light/dark box to examine whether the extinguished fear memory was reinstated. As shown in Figure 4 and Supplemental Figure 3, the tree shrews showed a small trend toward increased latency when it came to entering the dark box and reduced percentage of staying time in the dark box, implying reinstatement effects in the fear memory in this species.

## Discussion

In this study, we described an experimental paradigm that can successfully elicit the long-term memory of fear in tree shrews. Not surprisingly, tree shrews need a bigger arena compared to mice and rat, as well as a cover to prevent them from jumping out of the apparatus. In this study, conditioned stimulus is the context (light or dark side of the box). We monitored the activity of tree shrews in the light/dark box and applied voltage-induced foot shocks as stimulus US. The avoidance behavior, but not the freezing behavior, was used to index the establishment and extinction of fear memory. This was done largely because the natural defensive behavior of tree shrews to danger is flight rather than freezing. The range of stimulating currents that induced significant avoidance behavior (0.26–1.27 mA) was comparable to that used in fear conditioning for rodents (0.3–1.0 mA). The tree shrews requires 2–5 training sessions to establish the associated fear memory, and this was similar to the number required for mice and rats (1–10 training sessions, Chang et al., 2009; Tipps et al., 2014).

Similar to rodents, the tree shrews showed rapid formation of fear memory, characterized by long-term avoidance of the stimulating box during the post-training period (up to 51 days). Moreover, the established fear memory was reversed completely by only several rounds of reverse training, suggesting that tree shrews are very flexible in learning. Memory extinction in tree shrews is dependent on the intensity of the US. It can vary from no memory extinction at all for 51 days with 16 V stimulus with a 30 s ISI for 5 days, to complete extinction over 12 days with 12 V stimulus with a 60 s ISI for 2 days. This is in contrast with the relatively fast extinction timecourse for rodents, where memory extinction typically occurs immediately after the presentation of the CS without the US (Milad et al., 2006; Peña et al., 2013; Tan et al., 2015). For instance, Tan et al. reported in 2015 that the percentage of freezing in rats declines linearly from roughly 70 to 20% within 5 days during extinction training when foot shocks at 1.2 mA were used to induce conditional fear (Tan et al., 2015), suggesting species variability between rodents and the tree shrews. Furthermore, the extinguished fear memory is reinstated by a reminder stimulus (Figure 4), clearly indicating that extinction in tree shrews represents new learning rather than erasure.

Other forms of learning and memory have been studied in tree shrews. Hole-board learning and eight arm maze tasks have been utilized to investigate cognitive learning ability in these animals (Ohl and Fuchs, 1998; Bartolomucci et al., 2002; Takahashi, 2011; Shen et al., 2014). In 2011, researchers used an automatic trapping cage to study the one-trial captive conditioning model of learning and memory in tree shrews (Wang et al., 2011). Moreover, in 2013, researchers used a paired cage to examine the curative effect of clomipramine in subordinate tree shrews (Wang et al., 2013a). Compared with avoidance learning, other researchers have examined approaching behaviors associated with novel object recognition in this species (Khani and Rainer, 2012; Nair et al., 2014). In contrast to the avoiding learning task, which can be induced by less intensive training (2–5 sessions), tree shrews need five or more training sessions to form the memory of novelty preference. The retention for acquiring novelty preference lasts for at least 24 h, and whether or not such memory (and others) could last as longer as that in avoidance learning will be of interest in future investigations.

To our knowledge, models for studying the formation and extinction of fear memory have rarely been discussed in the literature. The tree shrew is a species that is closer to primates than rodents in evolutionary terms and is easily acquired in many countries. Thus, our results validate a conditioning avoidance paradigm in tree shrews, enriching the existing behavioral paradigms concerning the processes of visual perception, decision making, stress, and object novelty preference in this species (Petry et al., 1984; Ohl and Fuchs, 1998; Ohl et al., 1998; Callahan and Petry, 2000; Khani and Rainer, 2012; Nair et al., 2014). Due to the large differences in brain anatomy and circuitry between flies, fishes, rodents, and humans, it remains an open question that whether identical mechanisms are involved in fear memory formation and extinction in human and nonhuman animals. Previous studies show that the amygdala/hippocampus volumetric ratios of tree shrews is 0.41 ± 0.01 that is closer to that of adult human (~0.67), in contrast to that of rat (0.20 ± 0.03, Watson et al., 1992; Wang et al., 2013b). Comparing the gene encoding 5-hydroxytryptamine receptor 2A (5HT2A) that is implicated in the formation and extinction of fear memory (Campbell and Merchant, 2003; Ji and Suga, 2007; Biagioni et al., 2013; Zhang et al., 2013), the sequence of tree shrews shares 96.8% similarity with human and Macaca, while mouse and rat sequences share 95.3 and 95.1% respectively (Supplemental Figure 4). Thus, expanding those studies to tree shrews, a species closer to humans, would potentially help to elucidate these processes. Further studies are required to combine multidisciplinary methods to map the circuit mechanisms underlying memory formation and extinction in a tree shrew model.

### Conflict of interest statement

The authors declare that the research was conducted in the absence of any commercial or financial relationships that could be construed as a potential conflict of interest.
