# Supplementary material for: The formation and extinction of fear memory in tree shrews
Source: Front Behav Neurosci. 2015 Jul 29;9:204. doi: 10.3389/fnbeh.2015.00204 (PMC4518201; doi:10.3389/fnbeh.2015.00204)
Supplement: Supplementary file 1 [file DataSheet1.DOCX]

**Supplemental Information for Shang et al., "** **The Formation and Extinction of Fear Memory in Tree Shrews"**


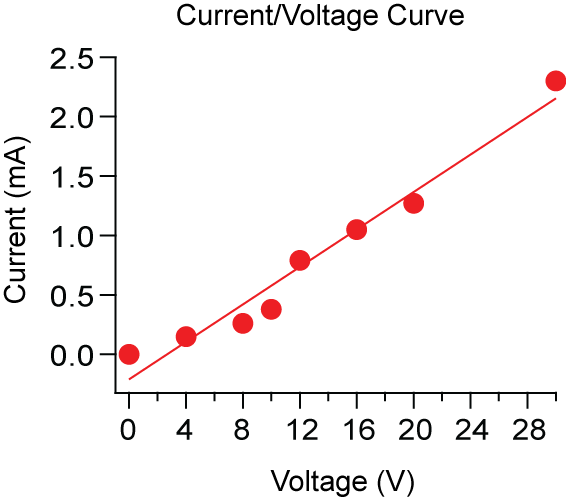


**Supplemental Figure 1.** The current-voltage curve of the light/dark apparatus.


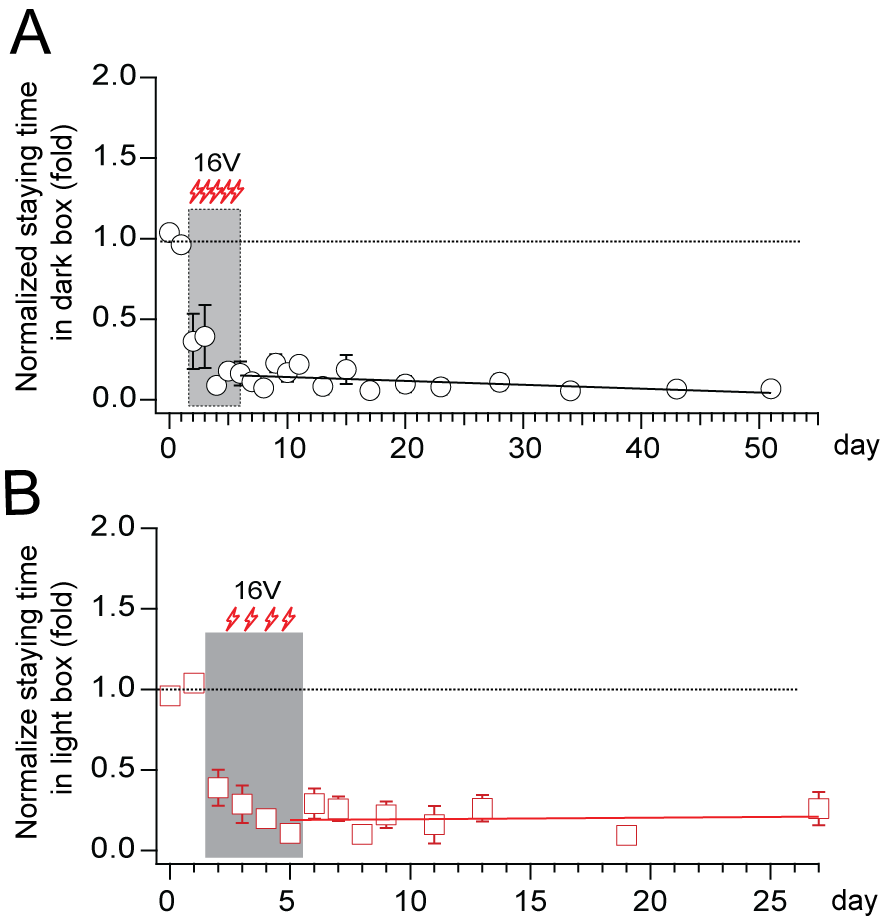


**Supplemental Figure 2.** No memory extinction after high-intensity stimulation.

**A** and **B.** There was no fear memory extinction after high-intensity stimulation (6 V, 5 times in **A** and 4 times in **B**) group, whenever the stimulus chamber was the dark box (**A**) or light box (**B**). All data were normalized to the pre-condition period in the stimulation chamber in A and B. All data were expressed as mean ± SEM (*** p < 0.001, n = 4).


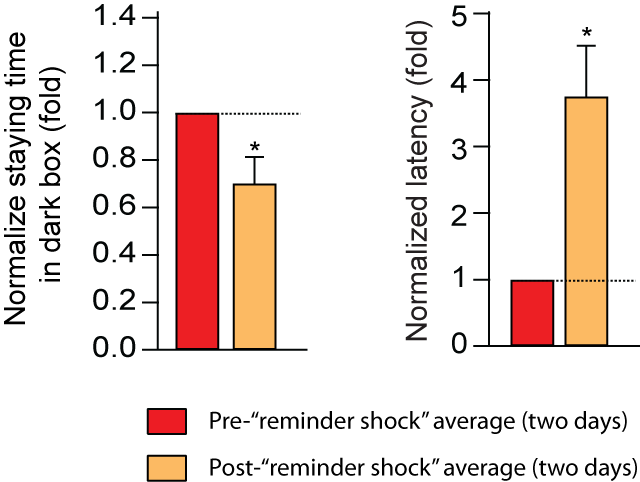


**Supplymental figure 3.** The comparision of staying time and latency to enter the dark box before and after the “reminder shock” in tree shrews. The 2-days average of staying time (left) and the latency to enter the dark box were calculated for both pre- and post-“reminder shock” periods. The average value of post-“reminder shock” period was normalized to that of pre-“reminder shock” period to reduce the [individual](http://cn.bing.com/dict/search?q=individual&FORM=BDVSP6&mkt=zh-cn) [variation](http://cn.bing.com/dict/search?q=variation&FORM=BDVSP6&mkt=zh-cn)s. All data were expressed as mean ± SEM (*p < 0.05, n = 7). The data were analyzed using a paired-samples t-test.


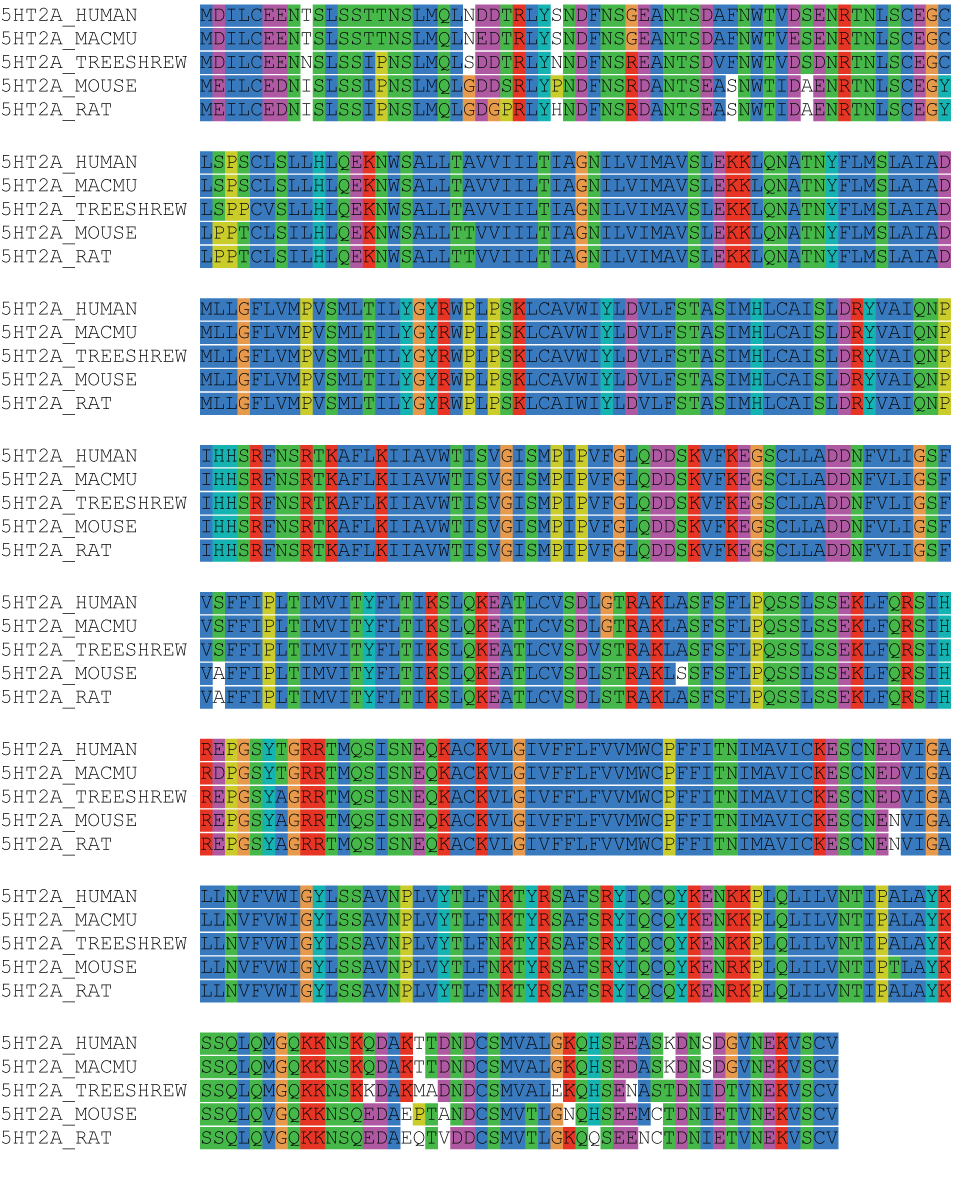


**Supplymental figure 4.** Comparision of the amino acid sequence of 5HT2A gene among human (P28223), macaca (P50128), mouse (P35363), rat (P14842) and tree shrews (ENSTBEG00000004580).
